# Ventilation distribution during spontaneous breathing trials predicts liberation from mechanical ventilation: the VISION study

**DOI:** 10.1186/s13054-024-05243-0

**Published:** 2025-01-07

**Authors:** Vorakamol Phoophiboon, Antenor Rodrigues, Fernando Vieira, Matthew Ko, Fabiana Madotto, Annia Schreiber, Nannan Sun, Mayson L. A. Sousa, Mattia Docci, Clement Brault, Luca S. Menga, Irene Telias, Thomas Piraino, Ewan C. Goligher, Laurent Brochard

**Affiliations:** 1 Unity Health Toronto, Keenan Centre for Biomedical Research, Li Ka Shing Knowledge Institute, 209 Victoria Street, Toronto, ON M5B 1T8 Canada; 2 Interdepartmental Division of Critical Care Medicine, University of Toronto, Toronto, ON Canada; 3 Division of Critical Care Medicine, Department of Medicine, Faculty of Medicine, Chulalongkorn University, Bangkok, Thailand; 4 Department of Anesthesiology, Intensive Care and Emergency, Fondazione IRCCS Ca’ Granda Ospedale Maggiore Policlinico, Milan, Italy; 5 Department of Critical Care Medicine, The First Affiliated Hospital of Shandong First Medical University & Shandong Provincial Qianfoshan Hospital, Jinan, Shandong China; 6 School of Medicine and Surgery, University of Milano-Bicocca, Monza, Italy; 7 Intensive Care Department, Amiens-Picardie University Hospital, Amiens, France; 8 Medical Surgical Neuro ICU, Toronto Western Hospital, University Health Network, Toronto, ON Canada; 9 Department of Anesthesia, McMaster University, Hamilton, ON Canada; 10 Division of Respirology, Department of Medicine, University Health Network, Toronto, ON Canada

**Keywords:** Ventilation-distribution, Electrical impedance tomography, Weaning

## Abstract

**Background:**

Predicting complete liberation from mechanical ventilation (MV) is still challenging. Electrical impedance tomography (EIT) offers a non-invasive measure of regional ventilation distribution and could bring additional information.

Research question.

Whether the display of regional ventilation distribution during a Spontaneous Breathing Trial (SBT) could help at predicting early and successful liberation from MV.

**Study design and methods:**

Patients were monitored with EIT during the SBT. The tidal image was divided into ventral and dorsal regions and displayed simultaneously. We explored the ventral-to-dorsal ventilation difference in percentage, and its association with clinical outcomes. Liberation success was defined pragmatically as passing SBT followed by extubation within 24 h without reintubation for 7 days. Failure included use of rescue therapy, reintubation within 7 days, tracheostomy, and not being extubated within 24 h after succesful SBT. A training cohort was used for discovery, followed by a validation cohort.

**Results:**

Among a total of 98 patients analyzed, 85 passed SBT (87%), but rapid liberation success occurred only in 40; 13.5% of extubated patients required reintubation. From the first minutes to the entire SBT duration, the absolute ventral-to-dorsal difference was consistently smaller in liberation success compared to all subgroups of failure (*p* < 0.0001). An absolute difference at 5 min of SBT > 20% was associated with failure of liberation, with sensitivity and specificity of 71% and 78% and positive predictive value 81% in a validation cohort.

**Conclusion:**

During SBT, a large ventral-to-dorsal difference in ventilation indicated by EIT may help to rapidly identify patients at risk of liberation failure.

**Supplementary Information:**

The online version contains supplementary material available at 10.1186/s13054-024-05243-0.

## Introduction

During weaning or separation or from mechanical ventilation (MV), spontaneous breathing trials (SBT) are employed as diagnostic tests to predict successful liberation from MV by assessing patient’s ability to breathe spontaneously. The accuracy of the SBT to predict extubation failure, however, is limited, whereas, at the opposite, reintubation is associated with a poor prognosis and high hospital mortality frequently around 30–40% [1]. In addition, 15–30% of patients are extubated without SBT, whereas a substantial number of patients succeeding an SBT are not extubated [2–5]. What really matters for the patient is the final and sustained successful liberation from mechanical ventilation and, focusing on SBT alone may not be sufficiently informative [6–8].

Monitoring ventilation in real time at the bedside has been made possible by the use of electrical impedance tomography (EIT), a non-invasive tool assessing regional distribution of ventilation [9, 10] Few studies have used EIT during SBT to correlate with weaning outcomes but they used complex parameters requiring off-line analyses [11–16].

Based on clinical observations of abnormal ventilation distribution in patients under MV [17], we wondered whether patterns of regional ventilation (distribution in ventral and dorsal regions) and pendelluft during spontaneous breathing could be associated with MV liberation outcome. We performed a prospective observational study to assess the association with regional distribution of ventilation using EIT in patients eligible for an SBT and MV liberation outcome. The amount of pendelluft using EIT and the morphology of the lung using ultrasound as well as other physiological parameters were assessed as secondary variables [18].

## Study design and methods

### Study population

This study was conducted at a tertiary academic critical care department in a medical-surgical and a neurotrauma ICU at St. Michael’s Hospital, Toronto, Ontario, Canada. It consecutively enrolled two cohorts of patients: a training cohort in October 2022-June 2023 and a validation cohort in August 2023-November 2023. The training cohort was designed for assessing variables that could predict rapid MV liberation. Subsequently, the validation cohort was used to validate the findings derived from the training cohort and additionally assess the morphology of the lung using ultrasound. This study obtained ethics approval from the Unity Health Toronto Research Ethics Boards board [REB #22–163]. Informed consent was obtained directly from patients or their substitute decision-makers (SDMs) and deferred consent was used when the patient was not competent and SDM not available.

Patients older ≥ 18 years, and having an indication for an SBT as per Toronto protocol [19] (see supplement) were enrolled: patient making inspiratory effort, PaO_2_/FiO_2_ ratio ≥ 200 mmHg and FiO_2_ ≤ 0.5, receiving < 0.2 mcg/kg/min of norepinephrine and on no > 1 vasoactive drug, with no concerns about intracranial pressure. Patients were excluded for contraindication to EIT placement or known palliative or end-of-life conditions. Our SBT procedure used zero CPAP and zero pressure support (as shown to be with T-piece, the most accurate predictor of post-extubation work of breathing) [20–22]. However, we also recorded data during the initial pressure support ventilation (PSV) period.

## EIT monitoring

A 16-electrode EIT was used to continuously monitor global and regional ventilation (PulmoVista500, Dräger Medical GmbH, Lübeck, Germany). The EIT belt was applied at 4th-5th intercostal space [9]. EIT data were analyzed offline using a dedicated software (EITdiag and EITanalysis). We selected 5–10 consecutive breaths after excluding breaths with artefacts (unstable signals from cough, suctioning or transient agitation).

### Absolute ventral-to-dorsal ventilation difference

**Fig. 1:**
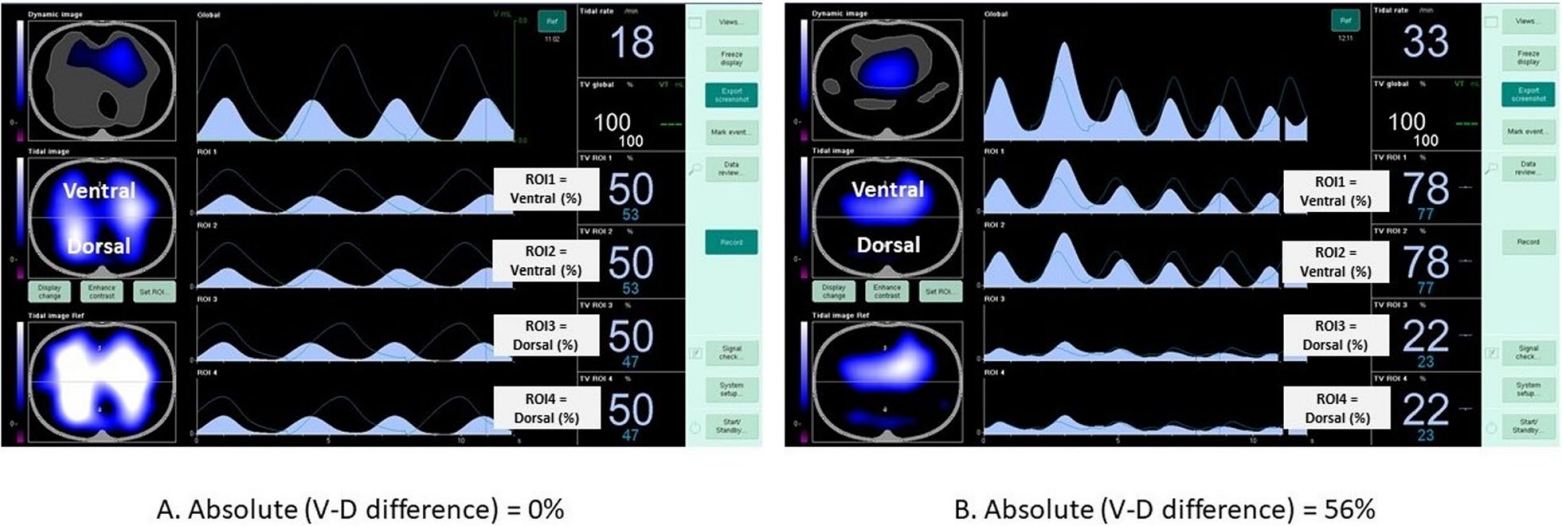
Representative examples of two electrical impedance tomography (EIT) screenshots allowing absolute ventral-to-dorsal difference calculation at the bedside. Of note, the ventral region was displayed in region of interest (ROI) 1 and duplicated in region of interest 2, and the dorsal region was displayed in region of interest 3 and duplicated in region of interest 4. These adjustments resulted in 2 customized regions of interest which displayed ventral versus dorsal ventilation distribution, as a percentage. **A** V% was 50 and D% was 50, the absolute ventral-to-dorsal difference was 0%. **B**, V% was 78 and D% was 22, and the absolute ventral-to-dorsal difference was 56%

The tidal image displayed in real time at the bedside on EIT screen was divided into two layers, ventral and dorsal as % of regional ventilation (V% and D%). Our primary measure was the absolute ventral-to-dorsal difference as illustrated in Fig. 1. We expressed all differences as positive values, whether the ventilation was predominantly ventral or dorsal.

### Pendelluft

Pendelluft was measured offline as the volume of gas moving from dorsal to ventral region. Pendelluft is an internal redistribution of gas within the lung, often from the anterior to the posterior part. This happens instead of a movement of fresh gas from outside and is therefore a part of ventilation which is wasted (supplement).

In addition, regional ventilation and pendelluft between the two lungs were also assessed.

## Lung ultrasound score (LUSS) and regional LUSS

We assessed lung morphology using LUSS and regional LUSS in the validation cohort (only) to understand the reasons underlying the differences in ventral and dorsal ventilation (supplement and eFigure 1).

## Data collection

Demographic data, comorbidities, indication for intubation, severity of illness (Simplified Acute Physiology Score II) and Sedation-Agitation Scale (SAS) were collected on admission. MV settings and the following variables were obtained throughout the study: rapid shallow breathing index (RSBI), airway occlusion at 100 ms (P0.1) and airway pressure negative swing during an end expiratory occlusion maneuver (ΔP_occ_) [6–8], blood pressure and heart rate. Following the completion of the SBT, outcomes were collected over 7-day, including time of extubation, of re-intubation, escalation in ventilatory support and tracheostomy, ICU length of stay and mortality.

MV liberation outcome (success or failure) was defined at 7 days after extubation as proposed [3, 21, 23, 24]. We defined rapid liberation success pragmatically as passing an SBT followed by a successful extubation the same or next day. All other situations were considered as early liberation failure and classified in four subgroups: 1) extubation followed by escalation to non-invasive ventilatory support (secondary requirement for high flow nasal cannula (HFNC) or non-invasive ventilation (NIV) as rescue therapy for post extubation occurrence of respiratory distress), 2) reintubation within 7 days, 3) SBT followed by clinical decision of tracheostomy and 4) not being extubated the same or next day after enrolment SBT.

## Study procedures

Measurements were obtained in a semi-recumbent position (30 degree of bed angle) at the following time points:

1. At baseline pressure-support level (before the SBT).
2. During the SBT: EIT recording was obtained 5 min for all patients and up to 30 min when they continued beyond the first 5 min (based on clinical tolerance). LUSS (validation cohort) were collected at the beginning (5 min) and the end (30 min) of SBT in the second phase of the study.

All patients received endotracheal suctioning before starting the measurement. The decision to perform SBT and determine the result were taken by the clinical teams blinded from the results of EIT and LUSS.

## Sample size

For the training cohort, no previous data existed and we calculated an initial sample size based on end-expiratory lung impedance (EELI) variation [12] because it had been reported to distinguish SBT outcome [12], but we recalculated a sample size of 52 patients using the absolute ventral-to-dorsal difference in MV liberation outcome from our pilot data, since we found this parameter to be potentially more interesting.

In the validation cohort, we validated prospectively the findings of the training cohort concerning the ventral-to-dorsal difference cut-off to predict MV liberation and assessed the value of LUSS; we calculated a sample size of 42. In total, a maximum of 110 patients with SBT were planned to be enrolled (training, n = 60 validation, n = 50, each cohort included 15% possible missing data). (supplement).

## Statistical analysis

We present the following analyses: 1. The absolute ventral-to-dorsal difference; 2. Their discriminative capacity to predict MV liberation, 3. The results of effort (ΔP_occ_), drive (P0.1) and pendelluft, 4. The results of LUSS and regional LUSS, 5. A multivariable regression model for the association with MV liberation.

Data are expressed as mean and SD or median IQR as indicated the normality of data using the Shapiro–Wilk’s test. Repeated continuous measurements on same patients at different time points during the SBT were analyzed using mixed-effects model with fixed effects of time point and liberation outcome along with a random effect for individual subject [25, 26]. The Holm-Šídák test was used for multiple comparison between estimated means at each time point [27]. Chi-square test was used to compare frequencies of categorical variables.

To identify the optimal cut-offs of ventral-to-dorsal difference that maximized sensitivity and specificity, receiver operating characteristic (ROC) curves [28] were assessed at each time point using the training cohort. The optimal cut-off was derived from SBT at 2 min. Two multivariable models were performed to assess whether the parameter was independently associated with the liberation outcome considering possible confounding factors: SAPS II for general severity, time from intubation to enrolment SBT for the duration of ventilation, intubation due to neurological conditions and the absolute ventral-to-dorsal difference as they both could influence MV liberation failure (supplement).

*P* values < 0.05 were considered statistically significant. Statistical analyses were conducted using GraphPad Prism 10.1.0 and R software version 4.2.2.

## Results

### Study population

A total of 110 mechanically ventilated patients with a clinical indication for SBT were enrolled, and 12 patients (5 patients from training and 7 from validation cohort) had to be excluded (6 transition to palliation; 4 repatriations; 1 tracheostomized before the SBT; 1 improper EIT belt position). Overall, the population analyzed consisted in 98 patients: a training cohort of 55 patients and a validation cohort of 43 patients.

**Fig. 2:**
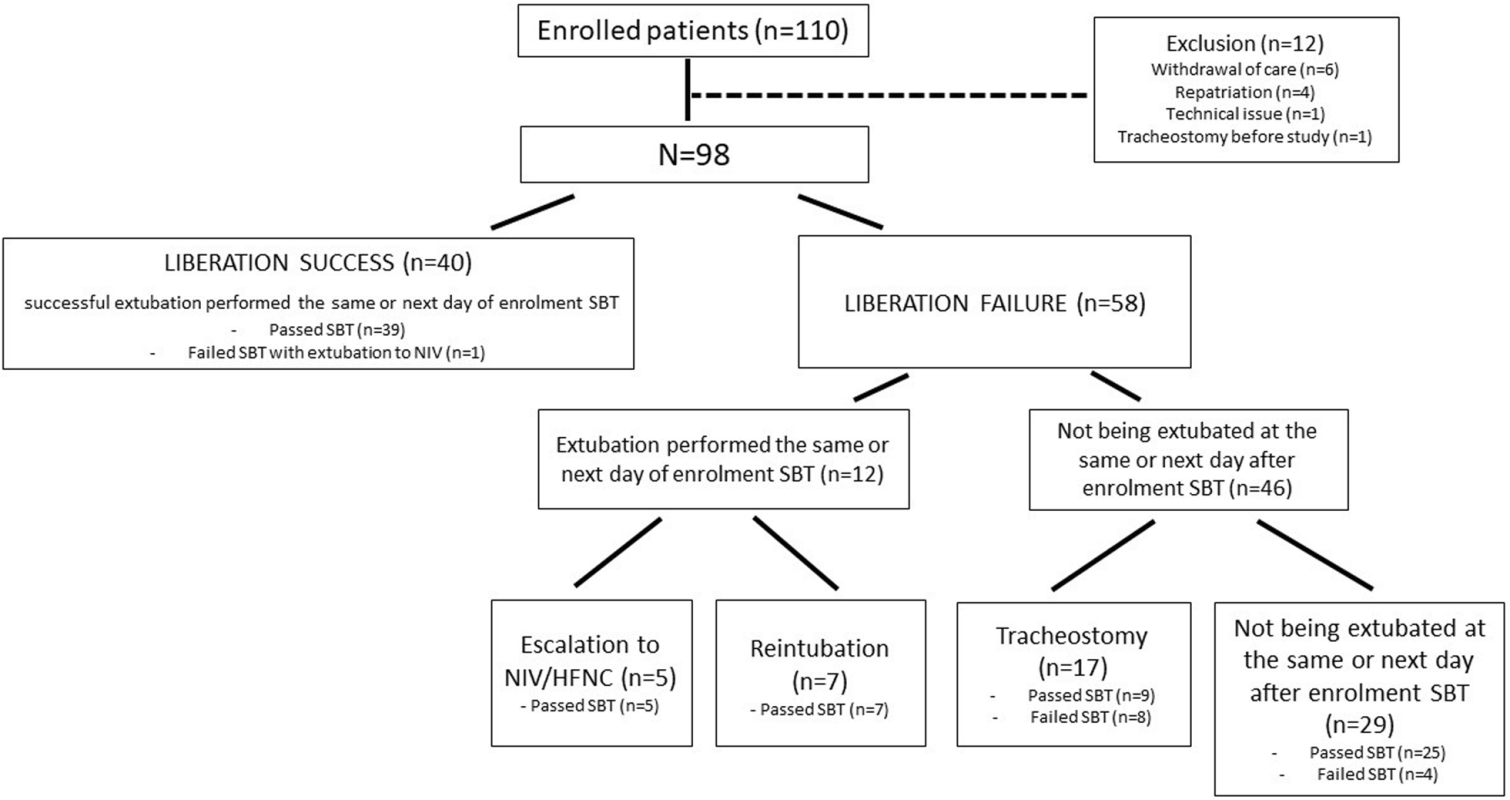
Patients’ flow chart in mechanical ventilation (MV) liberation outcome. One patient in liberation success group was extubated to NIV despite failing SBT because of previous use of home bilevel positive airway pressure. To note, a group of not being extubated at the same or the next day after enrolment SBT consisted of 4 patients with SBT failure at the enrolment, these patients were initially planned for tracheostomy but subsequently changed to extubation. For the 25 patients passing SBT but not being extubated at the same or next day after enrolment SBT, most of the patients were not extubated due to a concern of extubation failure related to a constellation of reasons (e.g., secretions, cough, fluid balance, frailty, etc.). In 2 patients, additional logistic reasons were present (waited for computed tomography, CT scan and non-urgent surgery)

Eighty-five patients (87%) passed the SBT as per respiratory therapists’ criteria. Although this was not formalized, extubation was not done immediately after SBT as patients were reconnected, waiting for the medical decision. Six patients failed early, i.e., 5 min after the SBT started while all others had 30 min of recordings. Early liberation success as defined above occurred in 40 (41%) and failure of all kinds in 58 (59%) patients, for the following reasons: 1) unplanned rescue escalation (non-prophylactic)(n = 5 (5%); all passed the SBT), 2) reintubation within 7 days (n = 7 (7%); they represented 13.5% of the 52 patients extubated at the same or next day of SBT and reintubation occurred after a median [interquartile range] of 4 [1–6] days), 3) medical decision for tracheostomy without attempting to extubate (n = 17 (17%); failed SBT, n = 8, passed SBT, n = 9, of note, these 9 patients were eventually separated from MV with a median of 14.5 [8.8–19.0] days, from 3 to 24 days after the study; and 4) being kept ventilated at least the next two days after the SBT (n = 29 (30%); passed SBT, n = 25; failed 4) (Fig. 2).

A total of 31 (32%) patients received prophylactic non-invasive ventilatory support (planned and used immediately after extubation, see supplement).

Characteristics of the population, MV settings and measurements during PSV and SBTs are shown in Tables 1 and e1. Patients with or without liberation success did not differ in terms of comorbidities and general severity at ICU admission. Neurological conditions were the most frequent indication for intubation (n = 48 or 49%, medical conditions 18 and neurosurgical conditions 30), followed by respiratory failure (31%). Neurological conditions were more frequent in failure patients (*p* = 0.007).

**Table 1:** Demographic and Clinical Characteristics of Cohorts, Stratified by Success and Failure in MV Liberation Outcome

	Overall study population (n = 98)			Training cohort (n = 55)			Validation cohort (n = 43)		
	Liberation success (n = 40)	Liberation failure (n = 58)	*P* value	Liberation success (n = 22)	Liberation failure (n = 33)	*P* value	Liberation success (n = 18)	Liberation failure (n = 25)	*P* value
Age, years	54 (16)	60 (16)	0.053	55 (17)	61 (19)	0.217	54 (16)	58 (16)	0.246
Sex, male, n (%)	24 (60)	40 (69)	0.360	13 (59)	25 (76)	0.190	11 (61)	15 (60)	0.941
Height, cm	173 (12)	172 (9)	0.605	172 (11)	173 (9)	0.884	172 (11)	170 (9)	0.508
Body mass index, kg/m^2^	25.5 [22.0–31.2]	25.6 [21.9–30.6]	0.767	25.5 [22.4–31.2]	24.3 [21.7–31.5]	0.582	25.6 [22.0–32.3]	28.1 [21.4–33.0]	0.951
SAPS II	38 (12)	42 (11)	0.066	37 (13)	42 (13)	0.166	38 (11)	43 (9)	0.146
SAS at enrolment, n (%)			0.269			> 0.999			0.217
SAS = 2	0	3 (5)		0	1 (3)		0	2 (8)	
SAS = 3	15 (38)	26 (45)		10 (45)	15 (45)		5 (28)	11 (44)	
SAS = 4	25 (63)	29 (50)		12 (55)	17 (52)		13 (72)	12 (48)	
Comorbidities									
Hypertension, n (%)	14 (35)	33 (57)	0.033	7 (32)	20 (61)	0.036	7 (39)	13 (52)	0.395
Diabetes, n (%)	7 (18)	16 (28)	0.247	4 (18)	10 (30)	0.230	3 (17)	6 (24)	0.560
Chronic lung disease, n (%)	13 (33)	10 (17)	0.080	8 (36)	6 (18)	0.097	5 (28)	4(16)	0.349
Congestive heart failure, n (%)	3 (8)	5 (9)	0.842	1 (5)	4 (12)	0.473	2 (11)	1 (4)	0.367
Renal failure, n (%)	3 (8)	11 (19)	0.111	0 (0)	8 (24)	0.015	3 (17)	3 (12)	0.663
Liver disease, n (%)	2 (5)	2 (3)	0.382	1 (5)	0 (0)	0.235	1 (6)	2 (8)	0.756
Active cancer, n (%)	2 (5)	3 (5)	0.970	2 (9)	1 (3)	0.372	0 (0)	2 (8)	0.219
Obesity – BMI ≥ 30 kg/m^2^, n (%)	14 (35)	14 (24)	0.252	7 (32)	7 (21)	0.529	7 (39)	7 (28)	0.521
Reason for intubation									
Respiratory failure, n (%)	16 (40)	14 (24)	0.094	8 (36)	9 (27)	0.475	8 (44)	5 (20)	0.085
Neurological conditions, n (%)	13 (33)	35 (60)	0.007	9 (41)	17 (52)	0.440	4 (22)	18 (72)	0.001
Cardiac failure, n (%)	4 (10)	4 (7)	0.581	2 (9)	3 (9)	0.999	2 (11)	1 (4)	0.367
Post-surgery, n (%)	7 (18)	5 (9)	0.257	3 (14)	4 (12)	0.961	4 (22)	1 (4)	0.066
Time from intubation to enrolment SBT, days	4 [3–8]	7 [5-11]	0.003	5 [3–8]	7 [6–11]	0.017	4 [3–9]	6 [514]	0.055
Total ICU LOS, days	8 [4–13]	20 [11–26]	< 0.001	9 [4–12]	21 [12–27]	< 0.001	7 [5–17]	18 [11–26]	0.002
ICU Mortality, n (%)	6 (15)	11 (19)	0.610	5 (23)	6 (18)	0.721	1 (6)	5 (20)	0.178

Definition of abbreviation: SAPS II = simplified acute physiology score II, SAS = Sedation-Agitation Scale, ICU LOS = intensive care unit length of stay

SAS 2 = very sedated (arouse to physical stimuli but does not communicate or follow command, may move spontaneously), SAS 3 = sedated (difficult to arouse but awakens to verbal stimuli or gentle shaking, follow simple commands but drifts off again), SAS 4 = calm and cooperative (calm, easily arousable, follows command) [44]

Notes: Continuous variables are shown as mean (SD) or median [IQR], and p-values are from t-test or Wilcoxon test. Categorical variables are presented as count (percentage), and p-values are from Chi-square or Fisher’s exact test as appropriate

Bedside respiratory variables of breathing pattern and effort-RSBI, P0.1 and ΔPocc did not differ. Time from intubation to enrolment SBT and ICU length of stay were longer in liberation failure compared to success.

## MV Liberation

### Absolute ventral-to-dorsal difference

**Fig. 3:**
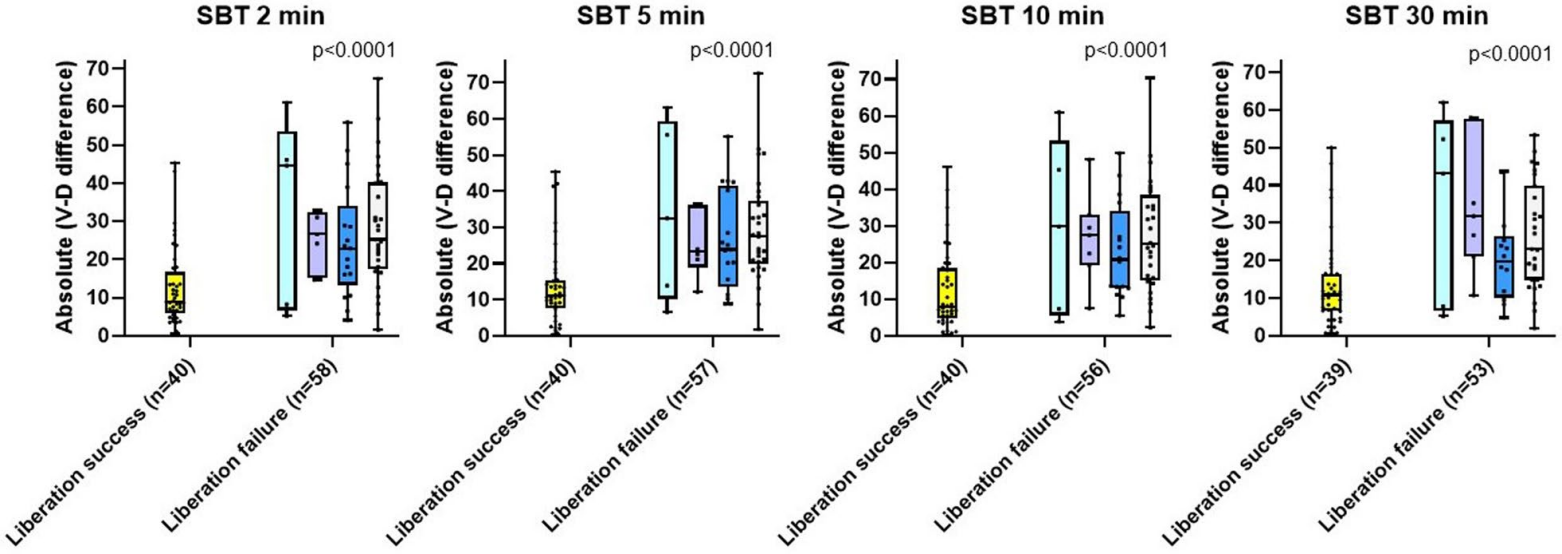
Absolute ventral-to-dorsal difference according to mechanical ventilation (MV) liberation outcome for the study population combined at 2, 5, 10, and 30 min of spontaneous breathing trial (SBT). Liberation success defined as a successful extubation performed the same or next day of enrolment SBT, yellow. For liberation failure, from left to right: extubation performed the same or next day of enrolment SBT followed by escalation in non-invasive ventilatory support, light blue; extubation performed the same or next day of enrolment SBT followed by reintubation, purple; tracheostomy, dark blue; not being extubated at the same or next day of enrolment SBT, grey. The p-value pertained to the comparison between the group that achieved liberation success and the groups that experienced failure

At each time point, the mean absolute ventral-to-dorsal difference was lower in liberation success than in all groups of liberation failure (escalation in support, reintubation, tracheostomy, and not being extubated at the same or next day) as shown in Fig. 3. The difference was already detected at the clinical PS level, *p* = 0.0005 and persisted until 30 min of SBT, *p* < 0.0001. The eFigure 2 shows a consistent result for the two cohorts combined at all time points indicating that the index was stationary.

When selecting only patients without neurological conditions (n = 50 of 98 patients), the absolute ventral-to-dorsal difference also differentiated patients between liberation success (n = 27) vs failure (n = 23), at the clinical PS level, *p* = 0.020 and persisted until 30 min of SBT, *p* ≤ 0.0003. (eFigure 3).

When looking only at the percentage of dorsal ventilation, a higher percentage of dorsal than ventral ventilation was present during the start of the SBT in liberation success. However, the difference between failure and success was smaller than for the absolute ventral-to-dorsal difference and of lower significance. (eFigure 4).

When looking at the mean absolute left-to-right difference, no difference existed according to MV liberation outcome. (eFigure 5).

### Discriminative Capacity to Predict MV Liberation

**Fig. 4:**
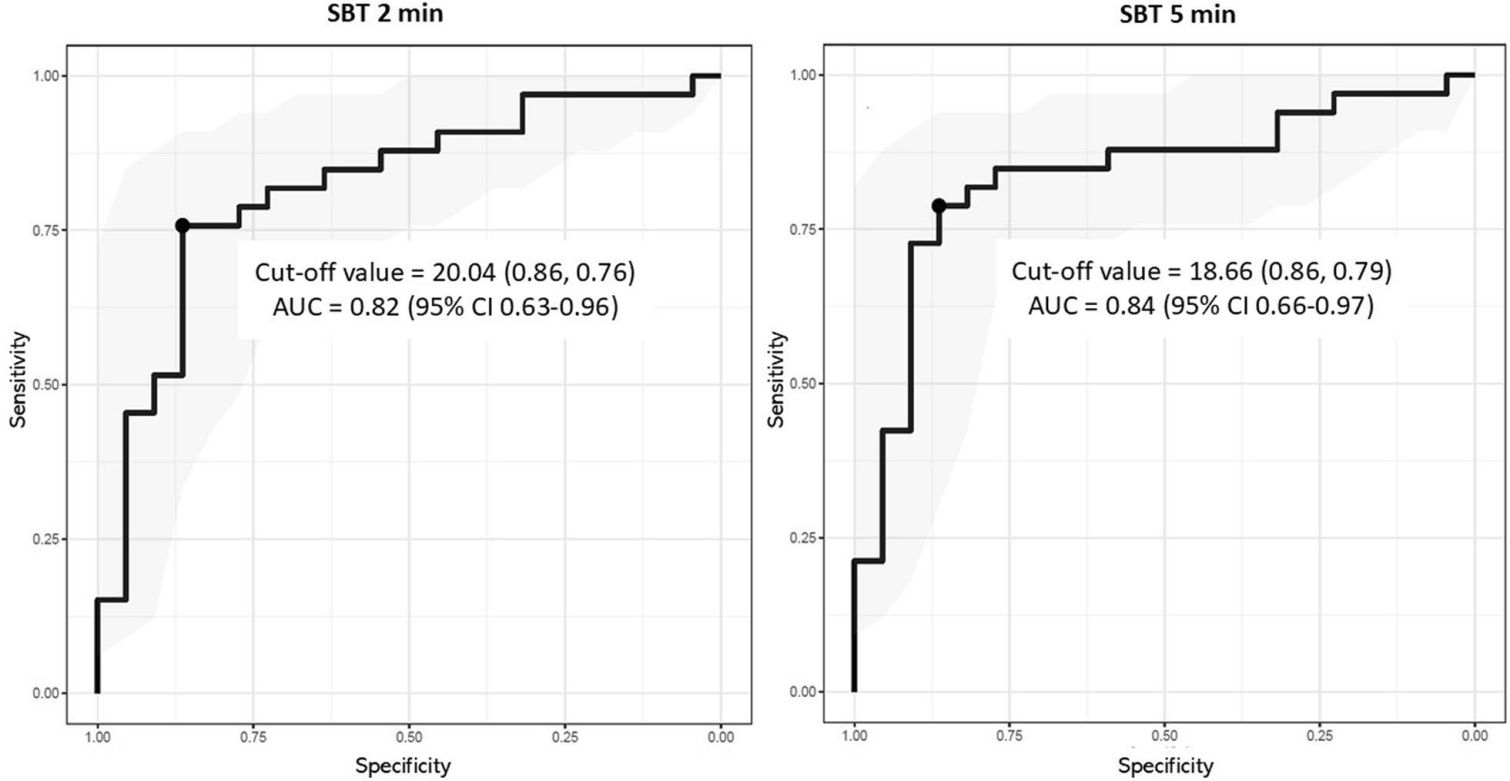
This figure displays receiver operating characteristic (ROC) curves and area under the ROC curve (AUC). The threshold values from the training cohort were absolute ventral-to-dorsal difference at 2, 5 min of spontaneous breathing trial (SBT) to identify the optimal cut-off value in mechanical ventilation (MV) liberation outcome

The training cohort consisted of 55 patients. Table 2 report the cut-off values for the absolute ventral-to-dorsal difference discriminating between success and failure. These values and sensitivity and specificity are shown by AUC in Fig. 4. The AUC during PSV was 0.73 while the AUCs at 2 to 5 min of SBT were from 0.80 to 0.84. Best thresholds varied slightly across time points, ranging from 18.64 to 22.99, with a 20.0% difference identified as the early cut-off at 2 min from the start of SBT.

**Table 2:** Discriminative Capacity of the Absolute Ventral-to-Dorsal Difference to Predict MV Liberation Outcome

	Training cohort (n = 55) ^ƚ^				Validation cohort using cut-off value of the absolute ventral-to-dorsal difference > 20 (n = 43)^*^			
SBT	Discriminative value	Sensitivity (%)	Specificity (%)	Area under the ROC curve with 95% Confidence Interval (CI)	Sensitivity (%)	Specificity (%)	Positive predictive value (%)	Negative predictive value (%)
PSV	22.99	55	86	0.73 (95% CI 0.54–0.89)	52	61	65	48
At 1 min	20.99	67	86	0.80 (95% CI 0.61–0.94)	52	83	68	56
At 2 min	20.04	76	86	0.82 (95% CI 0.63–0.96)	56	78	78	56
At 3 min	18.64	76	86	0.83 (95% CI 0.65–0.96)	64	78	80	61
At 4 min	20.77	70	86	0.83 (95% CI 0.66–0.96)	64	83	84	63
At 5 min	18.66	79	86	0.84 (95% CI 0.66–0.97)	71	78	81	67
At 10 min	22.10	61	91	0.82 (95% CI 0.64–0.95)	60	78	79	58
At 20 min	21.75	67	86	0.80 (95% CI 0.62–0.93)	60	72	76	59
At 30 min	22.70	62	86	0.75 (95% CI 0.55–0.91)	54	89	87	59

^ƚ^Values shown were derived from the prospective data set of training cohort (discovery cohort), comprising 22 successfully MV liberated patients and 33 patients in whom MV liberation failed

^*^Values shown were derived from the prospective data set of validation cohort (validated cohort), comprising 18 successfully MV liberated patients and 25 patients in whom MV liberation failed

Definition of abbreviations: MV = mechanical ventilation, PSV = pressure support ventilation, SBT = spontaneous breathing trial

A uniform cut-off value of > 20% in the absolute ventral-to-dorsal difference was used to predict MV liberation (higher values in failure; lower values in success) at each time point during clinical PS level and SBT in the validation cohort comprising 43 patients. Sensitivity, specificity, positive predictive value (PPV) and negative predictive value (NPV) are detailed in Table 2; specificity and PPV achieved at 2 to 5 min of SBT were 78% and 78%, and 78% and 81%, respectively.

Among patients who failed (n = 59), patients with predominant dorsal distribution were older (*p* = 0.014), more frequently intubated for respiratory failure (*p* < 0.001), and less often intubated for neurological conditions (*p* < 0.001) compared to those with a predominant ventral distribution (eTable 2).

### Multivariable regression model

Based on previous literature, on available data and on the number of events observed (one variable for ten events), the multivariable regression analysis used four independent variables (SAPS II, time from intubation to enrolment SBT, intubation due to neurological conditions and the absolute ventral-to-dorsal difference) associated with liberation outcome. An absolute ventral-to-dorsal difference greater than 20 or the ventral-to-dorsal difference as a continuous variable were independently associated with a greater risk of liberation failure, adjusted odds ratio for every % increase in VD difference 1.109 (1.059–1.175); *p* < 0.0001; Table 3).

**Table 3:** Multivariable regression model for the association with MV liberation outcome

Variable	Adjusted odds ratio (95% CI)	ß (95% CI)	Standard error	*P* value
*A. The absolute ventral-to-dorsal difference as a continuous variable*				
SAPS II	1.039 (0.994–1.090)	0.038 (− 0.006–0.086)	0.023	0.102
Time from intubation to enrolment SBT	1.194 (1.049–1.396)	0.177 (0.047–0.334)	0.072	0.014
Intubation due to neurological conditions	3.713 (1.317–11.360)	1.312 (0.275–2.439)	0.544	0.016
Absolute ventral-to-dorsal difference	1.109 (1.059–1.175)	0.104 (0.057–0.162)	0.026	< 0.0001
*B. Using the cut-off of 20% for the absolute ventral-to-dorsal difference*				
SAPS II	1.051 (1.005–1.104)	0.050 (0.005–0.099)	0.024	0.036
Time from intubation to enrolment SBT	1.195 (1.052–1.397)	0.179 (0.051–0.335)	0.072	0.013
Intubation due to neurological conditions	4.457 (1.555–14.300)	1.495 (0.441–2.660)	0.560	0.008
Absolute ventral-to-dorsal difference	12.980 (4.334–46.510)	2.563 (1.466–3.840)	0.599	< 0.0001

Definition of abbreviation: ß = estimated coefficient, 95% CI = 95% confidence interval, SAPS II = simplified acute physiology score II

## Secondary outcomes

### Effort, drive and pendelluft

RSBI, indexes of effort (ΔP_occ_) and of respiratory drive (P0.1) did not differ between the two groups (eTable 1 and eFigure 6).

Pendelluft was expressed as tidal volume %. The number of patients with pendelluft in the ventral-to-dorsal direction and between the two lungs was higher in the group with failure compared to success group but the amount of volume did not differ between groups. (eFigures 7 and 8) The presence of pendelluft had poorer predictive capacity than the absolute ventral-to-dorsal difference (eTable 3). The AUC fell to only 0.66 when adding the presence of pendelluft to the absolute ventral-to-dorsal difference (*p* = 0.005; eTable 4 and eTable 5).

### LUSS and regional LUSS

The LUSS and regional LUSS were measured in 43 patients of the validation cohort during PSV, first minutes and 30 min of SBT. There were no differences in the LUSS nor the regional LUSS between liberation success and failure. (eFigure 9) The median LUSS at the first minutes of SBT was 9 [0–14]. The addition of a LUSS > 13 did not improve the AUC with the absolute ventral-to-dorsal difference alone (0.76) whether measured at the beginning of SBT (0.63, *P* = 0.003) or at the end of SBT (0.59, *P* = 0.027; eTable 4 and eTable 5). All LUSS cut-off values had worse predictive capacity than the absolute ventral-to-dorsal difference (eTable 3). LUSS was not correlated with the absolute ventral-to-dorsal difference; both at the beginning of SBT, r = 0.027, *P* = 0.854; and at the end of SBT, r = 0.045, *P* = 0.775.

## Discussion

In this observational study, a difference in regional ventilation – expressed as an absolute ventral-to-dorsal difference measured by EIT- of less than 20% identified the patients going to be successfully liberated with 24 h, as early as in the first minutes of an SBT. Surprisingly the direction of inhomogeneity was not always a more ventral distribution as also indicated by ultrasound assessment, suggesting that the mechanism is not purely an absence of atelectasis. This novel finding suggests that a high heterogeneity of ventilation between ventral (non-dependent) and dorsal (dependent) regions of the lung during the SBT (no ventilatory support) is associated with a higher risk of early liberation failure, either identified by the clinicians (patients who were not extubated or underwent tracheostomy) or not identified by the clinicians and resulting in reintubation. These findings are intringuing and may help to better understand the complexity of the pathophysiology of weaning or liberation failure and could also have potential clinical predictive value for successful liberation from MV.

Our SBT protocol uses no support (equivalent to a T-piece) for reasons previously described [19]. Our findings, however, are not related to a specific SBT procedure because the absolute ventral-to-dorsal difference between MV liberation groups was already detected at PS 5 [5–8] and PEEP 5 [5–8] cmH_2_O, a setting used by some as SBT. Therefore, this absolute ventral-to-dorsal difference could be used at any different types of SBT protocol. The differences were slightly more pronounced without support (higher AUC). Last, differences were present as soon as the first minute of SBT, going against a progressive derecruitment observed along the SBT course.

We were interested by rapid and sustained MV liberation according to the various clinical scenarios occurring in the ICUs because this is what matters most for patients. Early liberation success was defined pragmatically as a successful SBT and a successful extubation (for seven days) performed at the same or next day of SBT. We regrouped liberation failure as all the other situations including SBT failure, extubation failure or patients passing their SBT but not extubated or tracheostomized by decision of the clinical team. The results are shown independently for all these categories and surprisingly they look very similar across the different types of failure, although number are smalls in some categories and should be interpreted cautiously.

In our study, 29% of patients passing the SBT were not extubated at the same or next day of enrolment. The clinical reasons for not extubating included poor level of consciousness, inadequate secretion clearance, and clinicians considering extubation unsafe, for instance in a neurosurgical context. A discrepancy between the result of the SBT and the decision for extubation is consistent with the findings of the WIND study, a large epidemiological study conducted in France, in which only 58% of the patients who passed the SBT were actually extubated directly [4]. This finding was also present in our previous multicenter study looking at sleep in the ICU [2]. Interestingly in the patients not extubated despite passing their SBT, based on clinical decision, distribution of ventilation looked similar to patients who were extubated but failed extubation.

A regional difference in ventilation between ventral and dorsal regions seems to convey important information. Previous studies have suggested that ventilation inhomogeneity (i.e., RVD index, GI index, Pendelluft) [11, 12, 15, 16] and lung de-recruitment (i.e., EELI) [11, 12] during SBT were associated with increased risk of weaning failure. However, regional ventilation distribution, a very simple index available at the bedside in real time (contrary to the previous indexes) has not been studied until now, whereas it seems to give information in the first minutes of SBT. In patients with healthy lungs, regional ventilation distribution is expected to be typically balanced between ventral and dorsal regions [29], while in lung injury the regional difference often increases [30]. Two previous studies have focused on this aspect uniquely analyzed by EIT although none has applied it during weaning. Yoshida et al. have shown that a predominance of dorsal ventilation with PEEP could be used as an incentive to reduce PEEP [17]. More recently, Iwata and colleagues explored the phenotype of ventilation patterns after surgery by utilizing EIT and showed an association of abnormal distribution with post-operative pulmonary complications [31]. Inhomogeneous ventilation patterns (either ventral or dorsal predominant) were associated with post operative pulmonary complications.

In our patients who failed MV liberation, patients with predominant dorsal ventilation were more frequently intubated for respiratory failure, while neurological condition was the most common reason in patients with ventral predominance. Ventral ventilation can be associated with atelectasis [32]. An increased use of extra-diaphragmatic inspiratory muscles could also lead to an increased ventral ventilation [33]. Conversely, a predominance of dorsal ventilation could be associated with increased diaphragmatic activity or high abdominal muscle use [34, 35]. We did not find a strict correlation with lung morphology [36]. Soummer et al. found that LUSS could predict extubation failure in patients passing an SBT [37]. However, we could not find significant differences in LUSS for liberation outcome. Several factors could explain the differences: our population and classification were different: we included both patients passing and failing the SBT in the failure group; patients receiving tracheostomy and not extubated at the same or next day were also considered failure in our study; we deemed liberation success after 7 days of extubation. Last, no previous study has used the ventral-to-dorsal difference with LUSS for the weaning/extubation outcome so we cannot make a real comparison.

Pendelluft is a pendular movement of gas between different lung regions without gas exchange. This phenomenon was described with regional heterogeneity in time constant (gas moves from faster lung regions towards slower ones) [38, 39]. We opted to calculate pendelluft focusing only on the last phase of expiration/first phase of inspiration in order to isolate the effect of the inspiratory effort on the intratidal shift of ventilation, without consideration to the additional role of different regional compliances and time constants. This approach is similar to studies showing differences during weaning, by Arellano et.al and Coppadoro et al. [15, 40]. Different pendelluft calculations have different objectives and can yield different results, some focusing on regional time constants and inspiratory delay [41] or calculating pendelluft from the entire breath [42]. Recently, the effect of pendelluft was studied during SBT and a high pendelluft occurrence was associated with SBT failure and reduction of CO_2_ elimination [15]. We observed a similar pendelluft incidence in our study (62%) than in previous studies (40–85%) [15, 40]. The number of patients having pendelluft in the failure group was larger than in the success group but we did not find a difference in the amount of pendelluft, in terms of volume, associated with liberation outcome. Interestingly, the number of patients having pendelluft occurring between the two lungs was also different between success and failure.

Our study has limitations. It was conducted in two ICUs at a single center. EIT is a new technology that is not yet available worldwide and may limit the applicability of our results. In our study, a high incidence of patients intubated for medical or surgical neurological conditions [43] may limit the generalizability of the findings, however, the subgroup analysis of patients without neurological conditions showed similar results regarding the absolute ventral-to-dorsal difference in liberation outcome. Although more sophisticated methods for assessing regional ventilation can involve offline analysis of ventilated regions, the absolute ventral-to-dorsal difference is simple and convenient for clinical use as it can be read directly at the bedside during ventilation. Last, we were unsure how to classify patients with rescue ventilatory support and we separated from those receiving planned HFNC or NIV. However, it is only a small group and keeping them in the success group would not change the results. Prophylactic non-invasive ventilatory support (immediately after extubation) is commonly used to prevent the risk of extubation failure in current practice based on perceived risks described in the literature.

## Conclusion

We have identified a novel finding in terms of regional ventilation distribution during SBT using the absolute ventral-to-dorsal difference, that may be used to help prognostication and optimize treatment to promote liberation success at the bedside.

## Supplementary Information

Additional file1 (DOCX 1855 KB)

## Data Availability

Most Data are available within the manuscript or supplementary information files.

## Abbreviations

P0.1: Airway occlusion at 100 miliseconds
ΔP_occ_: Airway pressure negative swing during an end expiratory occlusion manuever
EELI: End-expiratory lung impedance
EIT: Electrical impedance tomography
GI index: Global inhomogeneity index
HFNC: High flow nasal cannula
ICU: Intensive care unit
LUSS: Lung ultrasound score
MV: Mechanical ventilation
NPV: Negative predictive value
NIV: Non-invasive ventilation
PEEP: Positive end-expiratory pressure
PPV: Positive predictive value
PSV: Pressure support ventilation
RSBI: Rapid shallow breathing index
ROC: Receiver operating characteristic
RVD index: Regional ventilation delay index
REB: Research ethics board
SAPS II: Simplified acute physiology score II
SBT: Spontaneous breathint trial
SDM: Substitute decision maker
