# Supplementary material for: Ventilation distribution during spontaneous breathing trials predicts liberation from mechanical ventilation: the VISION study
Source: Crit Care. 2025 Jan 7;29:11. doi: 10.1186/s13054-024-05243-0 (PMC11705700; doi:10.1186/s13054-024-05243-0)
Supplement: Supplementary file 1 — Additional file1 (DOCX 1855 KB) [file 13054_2024_5243_MOESM1_ESM.docx]

**Supplementary Material**

**Methods**

**Unity Health Toronto Spontaneous Breathing Trials**

Procedure Overview

1. On a daily basis, the registered respiratory therapist (RRT) will perform a spontaneous breathing trial (SBT) Patient Safety Screen in consultation with the inter-professional patient care team. The RRT will assess and determine if an SBT is to be performed by responding to the following questions:

a. Does the patient make spontaneous inspiratory efforts (e.g. patient triggered breaths, diaphragm excursion)? If not, consider reducing minute ventilation (VE), or changing patient to a spontaneous mode to detect breathing efforts. NOTE: Ensure to return ventilator back to previous settings if no spontaneous inspiratory efforts are noted.

b. Is the patient’s PaO_2_/FiO_2_ ratio greater or equal to 200 and their FiO_2_ less than or equal to 0.50?

c. Is the PEEP less than or equal to 10 cmH_2_0?

d. Is the patient receiving less than 0.2 mcg/kg/min of norepinephrine or equivalent and on no more than 1 vasoactive drug?

e. In patients with acute brain injury the RRT needs to assess the following question: i. Is the patient’s neurological condition stable with no concerns about elevated intracranial pressure? (e.g. ICP has been stable and not requiring treatment for greater than 24 hours, this includes active CO_2_ control.)

2. If all of the above questions are answered YES, the RRT will conduct an SBT.

3. If any of the above questions are answered NO, the RRT will not perform an SBT. The RRT will continue mechanical ventilation and repeat the SBT safety screen in 24 hours or earlier as appropriate. The results of the SBT screen should be discussed with the inter-professional team.

4. To place the patient on an SBT the RRT will do the following:

a. All patients being weaned from ventilatory support will be monitored continuously by telemetry and pulse oximetry.

b. Ensure the patient is in a sitting or semi‐recumbent position.

c. Place the patient on the following ventilator settings: i. Mode: CPAP, ii. PEEP of 0 cmH_2_O, iii. FiO_2_ - unchanged from screening setting and iv. Ensure tube compensation functions are turned off, if applicable

d. Monitor closely for “tolerance” (see definition below). If SBT is not tolerated, return patient to ventilator settings that were previously set before initiation of the SBT trial or settings to meet physiological goals and patient comfort.

e. Two minutes after the SBT settings have been initiated the, RRT will calculate and document the RSBI of the patient

f. Note: If RSBI greater or equal to 110 active monitoring of patient for the next fifteen minutes is required.

g. The patient should be monitored on these settings for 30 to 60 minutes to determine if the SBT has been successful.

5. The RRT will determine if the patient tolerated the spontaneous breathing trial by assessing if the patient demonstrates any of the following failure criteria:

a. Respiratory rate of greater than or equal to 35 breaths per minute AND signs of respiratory distress for greater than 5 minutes. Note: respiratory distress may be characterized by anxiety, diaphoresis, accessory muscle use, tracheal tugging, and/or altered level of consciousness. An elevated respiratory rate in isolation may not indicate respiratory distress

b. A sustained SpO_2_ less than or equal to 88% for more than two minutes

c. A change (increase or decrease) in baseline heart rate of greater than 25%

d. A change (increase or decrease) in baseline mean arterial blood pressure of greater than 25%

6. If the patient displays any of the above symptoms or signs of intolerance to the SBT, the RRT will conclude that the patient failed the SBT. The RRT will restart mechanical ventilation as per previous settings or settings to meet physiological goals and patient comfort.

7. The RRT will document which SBT failure criteria the patient met. The results of the SBT should be discussed with the inter-professional team.

Note: If a patient has failed 3 consecutive SBTs, the RRT will communicate that to the inter-professional team in order to reassess weaning and care plan.

If the patient tolerates the SBT for 30 to 60 minutes without meeting any intolerance criteria, the RRT will place the patient on minimal ventilator support (e.g. PS of 5 cmH_2_O & PEEP of 5 cmH_2_O and will inform the inter-professional team that the patient has passed their SBT. At this time, the inter-professional team should consider extubation. If the patient passes the SBT trial and is not extubated, the RRT will document the reason for this decision.

**Non-invasive ventilatory support for prophylactic objective (planned and used immediately after extubation)’s criteria, the ICU’s accepted practice**

**Consider using NIV when patient may present with:**

1. Acute or chronic hypercapnic respiratory failure
2. Chronic obstructive pulmonary disease with/without acute exacerbation
3. Obesity
4. Acute pulmonary edema

**Consider using HFNC when:**

1. Concern of inadequate secretion clearance
2. Consider as high-risk patients (e.g., age, frailty, level of hypoxemia) for extubation*

*This will be identified by the clinical team at the time of extubation.

**EIT monitoring**

A 16-electrode EIT was used to continuously monitor global and regional ventilation (PulmoVista 500, Dräger Medical GmbH, Lübeck, Germany). The EIT belt was applied at 3^rd^-4^th^ or 4^th^ -5^th^ intercostal space as recommended.^1^ We selected 5-10 representative breaths after excluding breaths with artefacts related to patient’s movements, cough, or suctioning and noticed at the bedside. The analysis was performed using data recorded at specific time points of monitoring period: before SBT with PSV (PS level), during SBT (at 1, 2, 3, 4, 5, 10, 20, 30 minutes).

Because some patients had undetected elevated diaphragm (i.e., ileus, hepatosplenomegaly, ascites), we often placed the belt in the validation cohort at 3^rd^-4^th^ ICS and confirmed position by an ultrasound to avoid undetected elevated diaphragm. EIT data were analyzed offline using a dedicated software (EITdiag and EITanalysis, Dräger Medical GmbH, Lübeck, Germany).

**Pendelluft**

On the global EIT impedance trace over time, we looked first at the transition point from expiration to inspiration (T_0_). Pendelluft was detected when tidal impedance and/or volume difference between regions of interest (dorsal and ventral) demonstrated an earlier and later transition than T_0_, respectively. When the tracing of region of interest in dorsal region came earlier than T_0_, this was considered as an early inflation occurring during expiration which gained gas from the ventral region. Conversely, a late inflation of ventral region of interest was considered as losing gas during the early phase of the inspiration. We quantified pendelluft as the sum of the volumes of gas inspired from the early inflating regions of interest during global expiration and the late deflating regions of interest during global inspiration and when this amount was greater or equal to 2% of tidal volume.^2^ The same technique and criteria were used to identify the presence of the pendelluft occurring between left and right of the lungs.

**LUSS and Regional LUSS**

The LUSS is based on examination of 12 lung regions and has been proposed to assess lung aeration in mechanically ventilated patients.^3,4^ We measured the LUSS using a 2-4 MHz, convex transducer placed at the level of 6 lung regions delineated by the parasternal, anterior axillary, posterior axillary and paravertebral line on each side of the thorax. The LUSS is based on the regional aeration of each examined region, which is graded between 0 and 3.^5^ Quantitative LUSS (qLUSS) was used to grade the score in our study.^6^ The LUSS identified 0 as normal aeration – A lines and/or less than 3 of B lines; 1 as moderate loss of aeration – well-spaced B lines if ≥ 3, coalescent B lines and subpleural consolidations, occupying ≤ 50% of the pleura; 2 as severe loss of aeration - well-spaced B lines if ≥ 3, coalescent B lines or subpleural consolidations, occupying > 50% of the pleura; 3 as complete loss of aeration – tissue- like pattern, this score included a presence of pleural effusion with consolidation. The LUSS can vary between 0 (normally aerated lung) and 36 (totally consolidated lung). In our study, the regional LUSS and regional LUSS difference were created and analyzed to predict MV liberation outcome as well as the LUSS. The areas of interest of the regional LUSS difference are similar to the areas used for the absolute ventral-to-dorsal difference from the EIT.

We also calculated a regional LUSS difference as the difference of score between posterior and anterior regions of lung. Regional LUSS consists of the anterior (a sum of score at bilateral areas of 1, 2 and 3) and the posterior (a sum of score at bilateral areas of 4, 5, and 6) regions of the lung. The regional LUSS difference was the difference of score between posterior and anterior regions of lung.


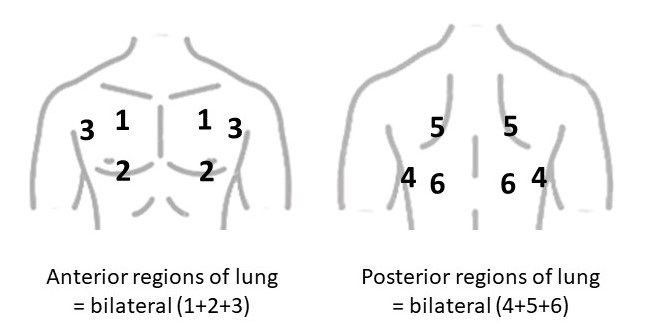


eFigure 1. The lung ultrasound score (LUSS) is the sum of bilateral (1+2+3+4+5+6) lungs. Anterior regions of lung are bilateral (1+2+3) lungs and posterior regions of lung are bilateral (4+5+6) lungs. The regional LUSS difference is the difference of score between posterior and anterior regions of lung.

**Sample Size**

For the training cohort, as no previous data existed on regional distribution and MV liberation outcome, we initially calculated a sample size based on 1) end-expiratory lung impedance (EELI) variation (changes of global ventilation during end expiratory phase) in the SBT outcome using Longhini et al.^7^; 2) we then recalculated a sample size using the absolute ventral-to-dorsal difference in MV liberation outcome from our pilot results (n=15) since we found this parameter to be potentially much more interestings. Accordingly, the sample size needed was 52 patients. On our first 15 patients, the absolute ventral-to-dorsal difference between patients with MV liberation success and failure was 16. Our targeted sample size was a maximum of 60 patients for the training cohort with adding 8 (15%) patients to account for potential missing data. This sample size was calculated considering a type I error of 5% and power of 99% to detect significant differences.

In the validation cohort, we wanted to validate prospectively the findings of the training cohort concerning the absolute ventral-to-dorsal difference cut-off value to predict MV liberation but also assess the value of LUSS. Our needed sample size was 42 patients based on data from Soummer et al.^3^, considering a mean LUSS of 19 in patients with extubation failure, compared to a mean LUSS of 10 in patients with extubation success at the end of SBT, with estimating standard deviation (SD) of 10 using Wan’s formula.^8^ This sample size considered a type I error of 5% and power of 80% and included 15% chance of missing data. Therefore, a maximum of 50 patients were our targeted sample size for the validation cohort.

We planned to enrol 110 patients with a clinical indication to perform an SBT (training cohort, n = 60 and validation cohort, n = 50).

**Statistical Analysis**

Data were expressed as mean and SD or median and interquartile range (IQR) as indicated the normality of data using the Shapiro-Wilk’s test. Repeated continuous measurements on same patients at different time points during the SBT were analyzed using mixed-effects model with fixed effects of time point and MV liberation outcome along with a random effect for individual subject. The Holm-Šídák test was used for multiple comparison between estimated means at each time point. Chi-square test was used to compare frequencies of categorical variables.

To identify the optimal cut-offs of absolute ventral-to-dorsal difference that maximized sensitivity and specificity, receiver operating characteristic (ROC) curves were assessed at each time point using data collected on the training cohort. The optimal cut-off of absolute ventral-to-dorsal difference was derived from SBT at 2 minutes.

To evaluate the predictive capacity of the absolute ventral-to-dorsal difference, the LUSS and the presence of pendelluft in predicting liberation failure, we employed 2x2 tables to calculate sensitivity, specificity, and positive and negative predictive value. The Area Under the Curve (AUC) was estimated, and DeLong’s test was employed to assess difference in predictive capacity between parameters. Regarding the LUSS, which was only assessed in the validation cohort, its predictive capacity was evaluated using various cut-offs (i.e., 9, 13, 15, 18), some of which were proposed in the existing literature.^3^ In addition, to assess the correlation between LUSS and the absolute ventral-to-dorsal difference, Pearson’s correlation test was used.

To assess whether the absolute ventral-to-dorsal difference was independently associated with the MV liberation outcome considering the effects of possible confounding factors: SASP II for general severity, time from intubation to enrolment SBT for the duration of ventilation, intubation due to neurological conditions and the absolute ventral-to-dorsal difference as they could influence with MV liberation failure. These 4 independent variables were selected as we had 98 patients with the outcome of interest occurring in 40 events and they could be able to address clinical benefits associated with MV liberation failure. Two multivariable models were performed on whole study population. In one model, the absolute ventral-to-dorsal difference was used as a continuous variable, while in the other, it was considered as a categorical variable based on the proposed cut-off. Both models included potentially confounding factors for MV liberation outcome determined from literature.

The patients who failed MV liberation were classified in two groups according to the predominance of the distribution (ventral or dorsal), and patients’ parameters (demographic, clinical and MV measurements at 2 minutes of SBT) were compared with appropriate statistical tests, in order to detect characteristics associated more to ventral or dorsal distribution.

P values < 0.05 were considered statistically significant. Statistical analyses were conducted using GraphPad Prism 10.1.0 and R software version 4.2.2.

**Results**

**Absolute ventral-to-dorsal difference in MV liberation outcome
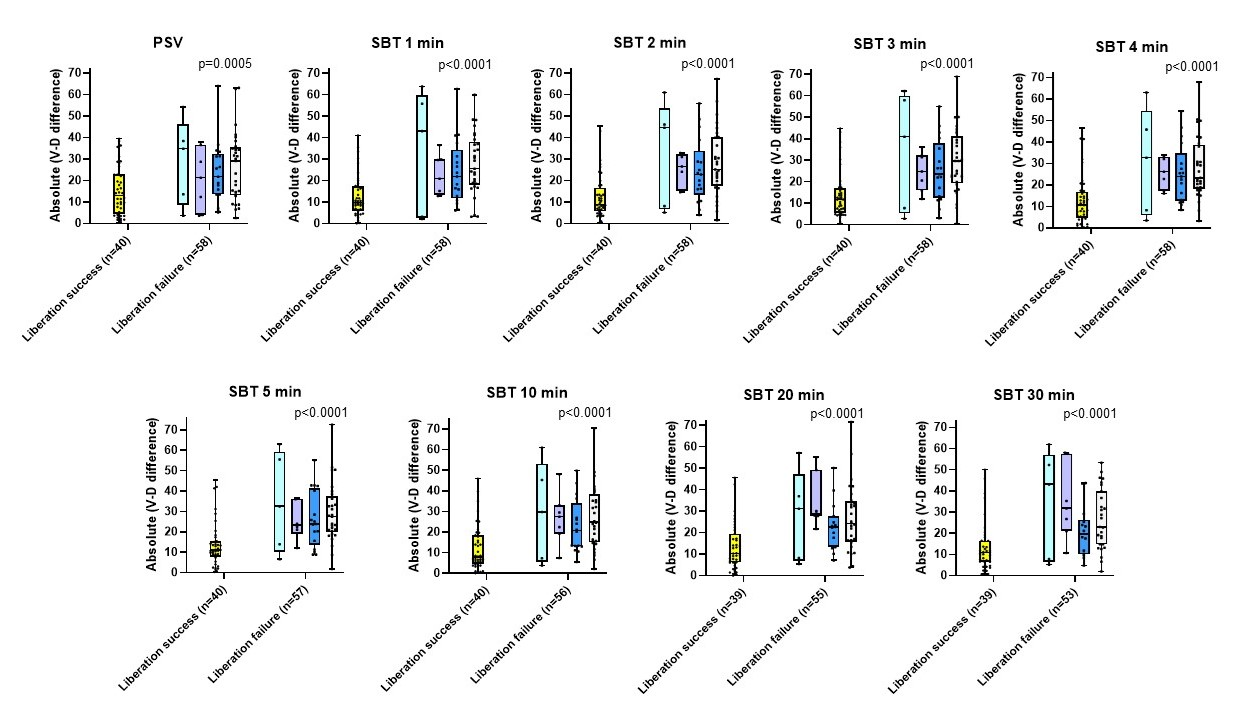
**

eFigure 2. Absolute ventral-to-dorsal difference according to mechanical ventilation (MV) liberation outcome for the study population combined at pressure support level (PSV), and 1, 2, 3, 4, 5, 10, 20, and 30 minutes of spontaneous breathing trial (SBT). Liberation success defined as a successful extubation performed the same or next day of enrolment SBT, yellow. In liberation failure, from left to right: extubation performed the same or next day of enrolment SBT followed by escalation in non-invasive ventilatory support, light blue; extubation performed the same or next day of enrolment SBT followed by reintubation, purple; tracheostomy, dark blue; not being extubated at the same day or the next day of enrolment SBT, grey. The p-value pertained to the comparison between the group that achieved liberation success and the groups that experienced failure.

**
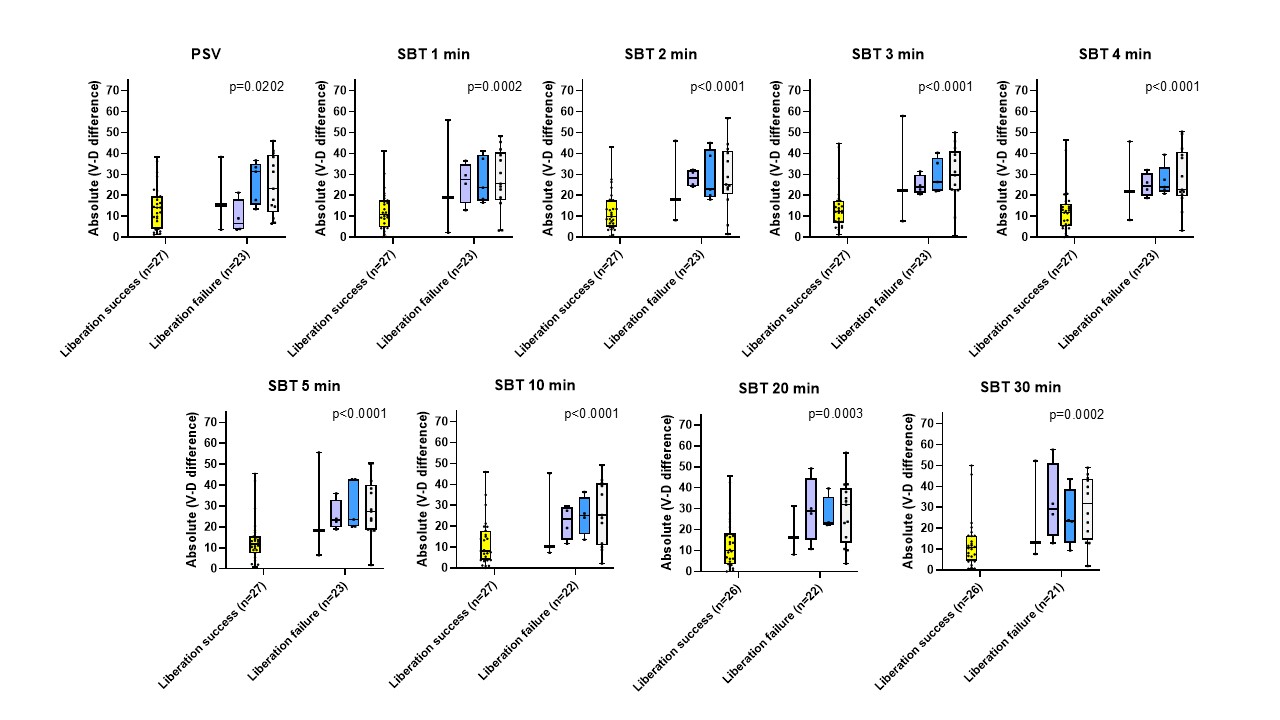
**

eFigure 3. Absolute ventral-to-dorsal difference according to mechanical ventilation (MV) liberation outcome for the patients without neurological conditions at pressure support level (PSV), and 1, 2, 3, 4, 5, 10, 20, and 30 minutes of spontaneous breathing trial (SBT). Liberation success defined as a successful extubation performed the same or next day of enrolment SBT, yellow. In liberation failure, from left to right: extubation performed the same or next day of enrolment SBT followed by escalation in non-invasive ventilatory support, first bar; extubation performed the same or next day of enrolment SBT followed by reintubation, purple; tracheostomy, dark blue; not being extubated at the same day or the next day of enrolment SBT, grey. The p-value pertained to the comparison between the group that achieved liberation success and the groups that experienced failure.

**Regional Distribution of Ventilation**


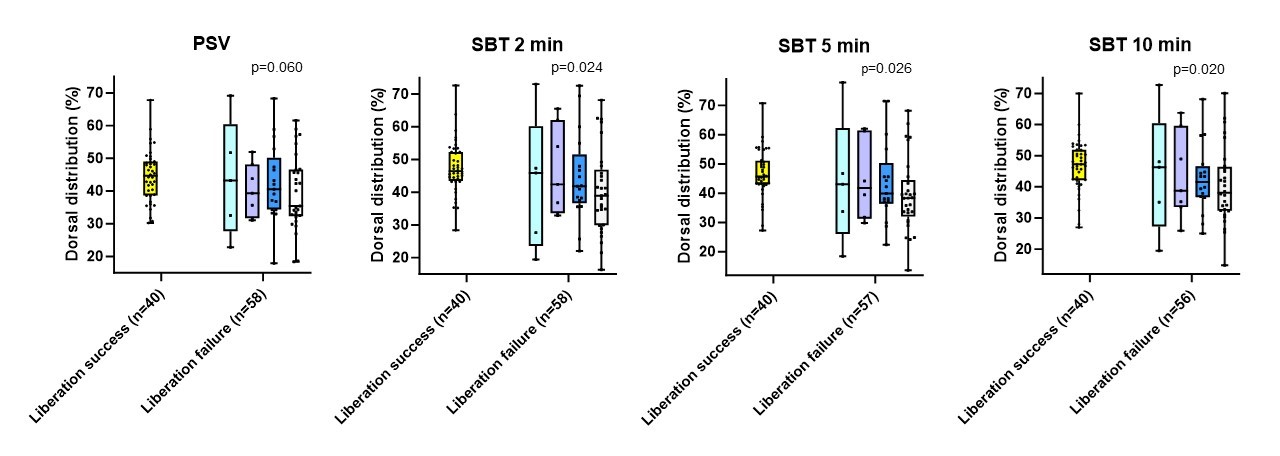


eFigure 4. Dorsal distribution (%) at pressure support ventilation (PSV), and 2, 5, and 10 minutes of spontaneous breathing trial (SBT). Liberation success defined as a successful extubation performed the same or next day of enrolment SBT, yellow. In liberation failure, from left to right: extubation performed the same or next day of enrolment SBT followed by escalation in non-invasive ventilatory support, light blue; extubation performed the same or next day of enrolment SBT followed by reintubation, purple; tracheostomy, dark blue; not being extubated at the same day or the next day of enrolment SBT, grey. The p-value pertained to the comparison between the group that achieved liberation success and the groups that experienced failure.

**
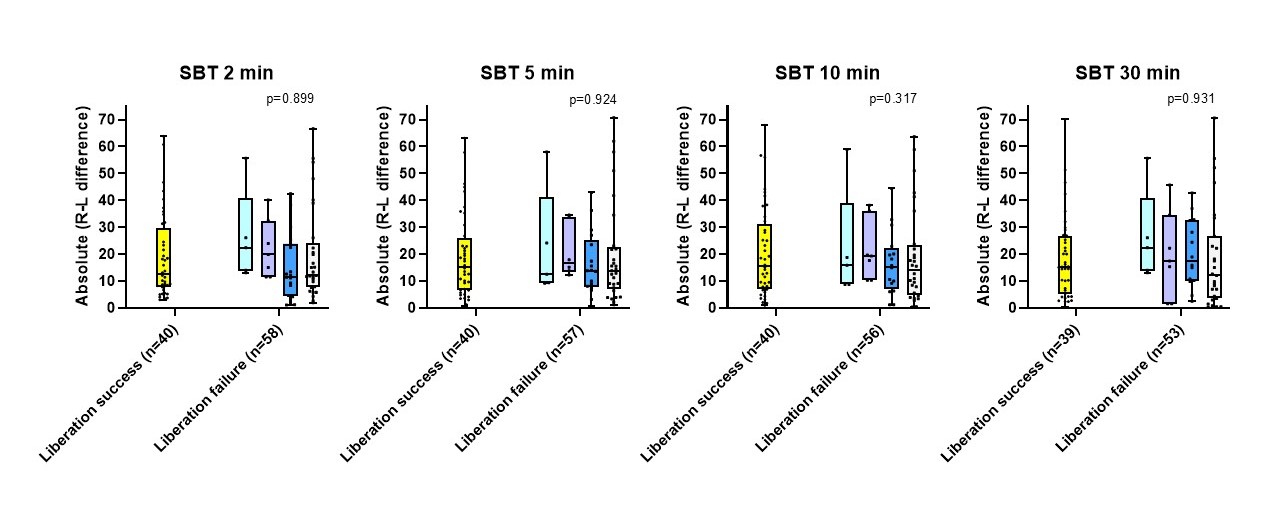
**

eFigure 5. Absolute left-to-right difference according to mechanical ventilation (MV) liberation outcome for the study population combined at 2, 5, 10, and 30 minutes of spontaneous breathing trial (SBT). Liberation success defined as a successful extubation performed the same or next day of enrolment SBT, yellow. In liberation failure, from left to right: extubation performed the same or next day of enrolment SBT followed by escalation in non-invasive ventilatory support, light blue; extubation performed the same or next day of enrolment SBT followed by reintubation, purple; tracheostomy, dark blue; not being extubated at the same day or the next day of enrolment SBT, grey. The p-value pertained to the comparison between the group that achieved liberation success and the groups that experienced failure.

**Effort, Drive and Pendelluft**


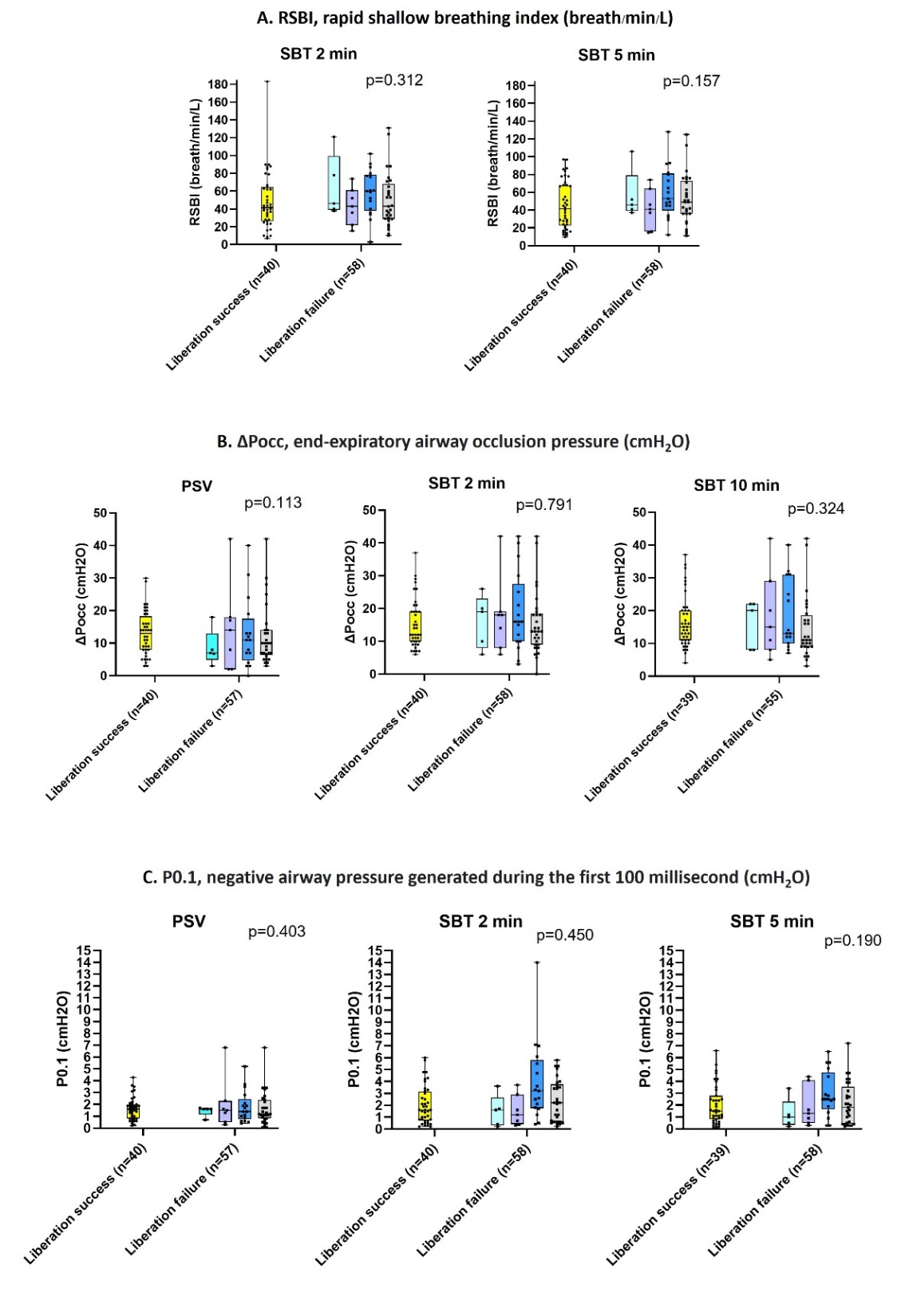


eFigure 6. A. rapid shallow breathing trial (RSBI) in mechanical ventilation (MV) liberation outcome for the study population at 2 and 5 minutes of spontaneous breathing trial (SBT). B. end-expiratory airway occlusion pressure (ΔPocc) in MV liberation outcome for the study popoluation at pressure support ventilation (PSV), and 2 and 10 minutes of SBT. C. negative airway pressure generated during the first 100 millisecond in MV liberation outcome for the study population at PSV, and 2 and 5 minutes of SBT. Liberation success defined as a successful extubation performed the same or next day of enrolment SBT, yellow. In liberation failure, from left to right: extubation performed the same or next day of enrolment SBT followed by escalation in non-invasive ventilatory support, first bar; extubation performed the same or next day of enrolment SBT followed by reintubation, second bar; tracheostomy, dark blue; not being extubated at the same day or the next day of enrolment SBT, grey. The p-value pertained to the comparison between the group that achieved liberation success and the groups that experienced failure.


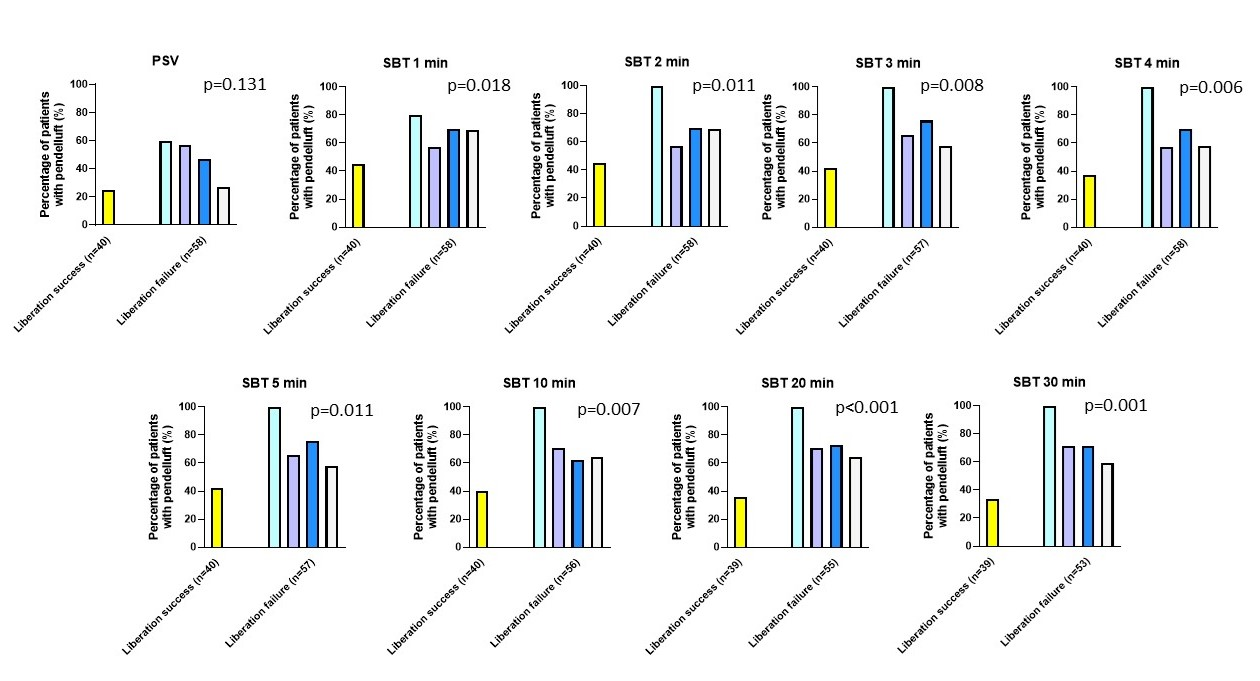


eFigure 7**.** Percentage of patients with pendelluft in mechanical ventilation (MV) liberation outcome at pressure support level (PSV), and 1, 2, 3, 4, 5, 10, 20, and 30 minutes of spontaneous breathing trial (SBT). Liberation success defined as a successful extubation performed the same or next day of enrolment SBT, yellow. In liberation failure, from left to right: extubation performed the same or next day of enrolment SBT followed by escalation in non-invasive ventilatory support, light blue; extubation performed the same or next day of enrolment SBT followed by reintubation, purple; tracheostomy, dark blue; not being extubated at the same day or the next day of enrolment SBT, grey. The p-value pertained to the comparison between the group that achieved liberation success and the groups that experienced failure.


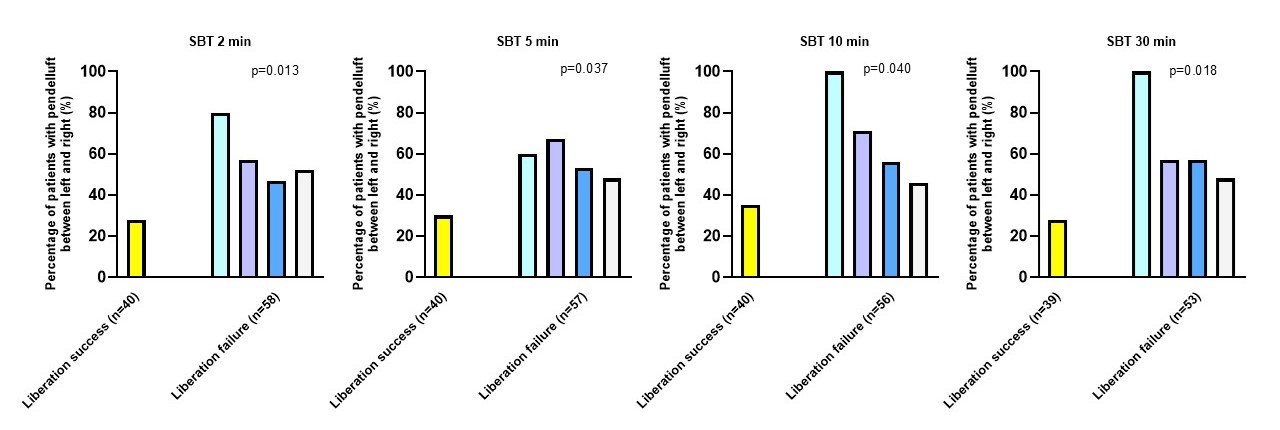


eFigure 8**.** Percentage of patients with pendelluft between left and right in mechanical ventilation (MV) liberation outcome at 2, 5, 10, and 30 minutes of spontaneous breathing trial (SBT). Liberation success defined as a successful extubation performed the same or next day of enrolment SBT, yellow. In liberation failure, from left to right: extubation performed the same or next day of enrolment SBT followed by escalation in non-invasive ventilatory support, light blue; extubation performed the same or next day of enrolment SBT followed by reintubation, purple; tracheostomy, dark blue; not being extubated at the same day or the next day of enrolment SBT, grey. The p-value pertained to the comparison between the group that achieved liberation success and the groups that experienced failure.

**LUSS and Regional LUSS**


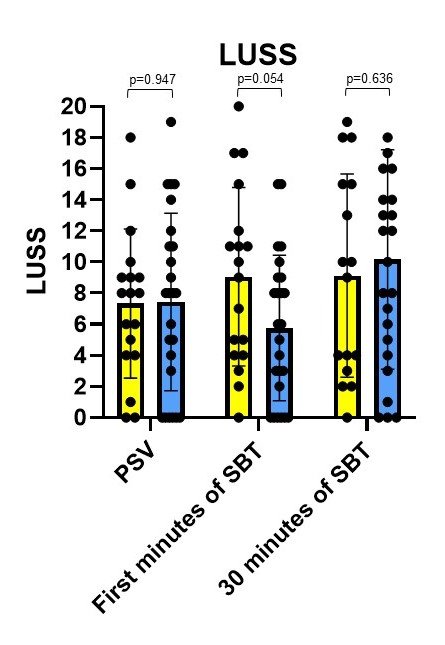


eFigure 9. Lung ultrasound score (LUSS) at pressure support ventilation (PSV), first minutes of spontaneous breathing trail (SBT) and 30 minutes of SBT in mechanical ventilation (MV) liberation outcome. Liberation success (n=18) showed in yellow bar and liberation failure (n=25) showed in dark blue bar during PSV, early minutes of SBT and 30 minutes of SBT.

eTable 1. MV Settings and Measurements during PSV and SBT of Study Population and Cohorts, Stratified by Success and Failure in MV Liberation Outcome.

|  | Overall study population (n=98) | | |  | | |  | | |
| --- | --- | --- | --- | --- | --- | --- | --- | --- | --- |
|  |  |  |  | Training cohort (n=55) | | | Validation cohort (n=43) | | |
|  | Liberation success (n=40) | Liberation failure (n=58) | P-value | Liberation success (n=22) | Liberation failure (n=33) | P-value | Liberation success (n=18) | Liberation failure (n=25) | P-value |
| **MV settings before SBT** | | | | | | | | | |
| PSV, cmH_2_O | 5 [5-8] | 5 [5-8] | 0.099 | 5 [5-8] | 5 [5-8] | 0.357 | 5 [5-6] | 5 [5-8] | 0.145 |
| PEEP, cmH_2_O | 5 [5-8] | 5 [5-8] | 0.584 | 5 [5-8] | 8 [5-9] | 0.221 | 5 [5-8] | 5 [5-8] | 0.586 |
| FiO_2_, % | 33 (6) | 32 (5) | 0.876 | 34 (7) | 34 (5) | 0.971 | 31 (6) | 32 (7) | 0.536 |
| **Measurements during PSV and SBT** | | | | | | | | | |
| RSBI, breath/min/L  SBT 2 min  SBT 5 min | 41 [26-64]  41 [22-68] | 50 [34-72]  49 [36-73] | 0.312  0.157 | 42 [36-73]  51 [29-54] | 56 [37-76]  48 [38-76] | 0.467  0.473 | 33 [17-56]  26 [17-56] | 63 [40-121]  51 [27-68] | 0.363  0.184 |
| ΔPocc, cmH_2_O  PSV  SBT 2 min  SBT 10 min | 13 [8-18]  12 [10-19]  15 [11-20] | 10 [6-14]  14 [10-19]  12 [9-22] | 0.113  0.791  0.324 | 14 [7-21]  16 [10-22]  15 [10-25] | 8 [5-13]  14 [8-18]  12 [9-21] | 0.054  0.262  0.258 | 11 [8-16]  12 [10-16]  15 [11-19] | 12 [7-17]  18 [11-25]  15 [9-28] | 0.835  0.122  0.860 |
|  | Overall study population (n=98) | | | Training cohort (n=55) | | | Validation cohort (n=43) | | |
|  | Liberation success (n=40) | Liberation failure (n=58) | P-value | Liberation success (n=22) | Liberation failure (n=33) | P-value | Liberation success (n=18) | Liberation failure (n=25) | P-value |
| P0.1, cmH_2_O (all values)  PSV  SBT 2 min  SBT 5 min | 1.6 [0.8-2.0]  1.6 [0.8-3.2]  1.5 [0.8-2.8] | 1.4 [0.8-1.9]  2.2 [0.7-3.8]  2.1 [0.6-3.9] | 0.403  0.190  0.450 | 1.7 [0.8-2.0]  0.8 [0.4-2.5]  1.0 [0.4-3.4] | 1.2 [0.6-2.2]  1.0 [0.5-2.4]  0.9 [0.4-2.3] | 0.576  0.704  0.775 | 1.6 [0.8-2.2]  2.0 [1.5-4.1]  1.9 [1.5-2.7] | 1.5 [1.1-1.9]  3.5 [2.6-4.6]  2.8 [2.3-4.2] | 0.826  0.026  0.025 |
| P0.1 (only on pressure trigger), cmH_2_O | (n=26) | (n=37) |  | (n=8) | (n=12) |  | (n=18) | (n=25) |  |
| PSV  SBT 2 min  SBT 5 min | 1.7 [1.3-2.0]  2.4 [1.6-4.1]  2.4 [1.5-4.1] | 1.5 [1.1-2.2]  3.5 [2.3-4.9]  2.8 [2.1-4.4] | 0.627  0.021  0.104 | 1.7 (0.2)  3.1 (1.2)  3.8 (1.7) | 1.8 (1.0)  4.0 (2.1)  3.7 (1.7) | 0.825  0.299  0.779 | 1.6 [0.8-2.2]  2.0 [1.5-4.1]  1.9 [1.5-2.7] | 1.5 [1.1-1.9]  3.5 [2.6-4.6]  2.8 [2.3-4.2] | 0.826  0.026  0.025 |
| MV, L/min  PSV  SBT 2 min  SBT 5 min | 8.7 (2.1)  9.4 (3.2)  9.1 (3.3) | 9.2 (2.7)  10.0 (3.2)  9.9 (3.2) | 0.398  0.327  0.205 | 9.0 (2.3)  9.5 (2.9)  9.4 (3.3) | 9.5 (3.0)  10.3 (3.3)  9.7 (2.9) | 0.568  0.372  0.651 | 8.3 (1.9)  9.2 (3.6)  8.8 (3.3) | 8.7 (2.0)  9.6 (3.2)  10.8 (3.6) | 0.534  0.654  0.188 |

Definition of abbreviations: PSV = pressure support ventilation, SBT = spontaneous breathing trial, MV = mechanical ventilation, PSV = pressure support ventilation, PEEP = positive end expiratory pressure, FiO_2_ = fraction of inspired oxygen, ΔPocc = end-expiratory airway occlusion pressure, RSBI = rapid shallow breathing index, P0.1 = negative airway pressure generated during the first 100 millisecond, SBT = spontaneous breathing trial. Continuous variables are shown as mean (SD), median [IQR] or count (percentage) as appropriate.

eTable 2. Demographic, Clinical Characteristics and MV Measurements at 2 Minutes of SBT

in MV Liberation Failure, Comparing Patients with A Predominant Ventral versus Dorsal

Distribution.

|  | MV liberation failure (n=58) | | |
| --- | --- | --- | --- |
|  | A predominance of ventral distribution (n=46) | A predominance of dorsal distribution (n=12) | P-value |
| Age, years | 58 [47-69] | 74 [63-77] | **0.014** |
| Sex, male, n (%) | 32 (70) | 8 (67) | 0.847 |
| Body mass index, kg/m^2^ | 24.0 [21.4-32.2] | 27.7 [24.6-29.7] | 0.451 |
| SAPS II | 42 (11) | 41 (12) | 0.674 |
| Reason for intubation:  Respiratory failure, n (%)  Neurological conditions, n (%)  Cardiac failure, n (%)  Post-surgery, n (%) | 6 (13)  33 (72)  4 (9)  3 (7) | 8 (67)  2 (17)  1 (8)  1 (8) | **<0.001**  **<0.001**  0.968  0.825 |
| RSBI at 2 minutes of SBT, breath/min/L | 43.5 [32.3-66.5] | 60.5 [38.5-88.0] | 0.104 |
| ΔPocc at 2 minutes of SBT, cmH_2_O | 14 [10-20] | 15 [7-18] | 0.765 |
| P0.1 at 2 minutes of SBT, cmH_2_O | 2.4 [0.7-3.6] | 1.5 [0.5-5.0] | 0.605 |
| Time from intubation to enrolment SBT, days | 7 [5-12] | 6 [5-10] | 0.750 |
| Total ICU LOS, days | 19 [11-27] | 21 [12-25] | 0.958 |
| ICU Mortality, n (%) | 7 (15) | 4 (33) | 0.154 |

Definition of abbreviations: MV= mechanical ventilation, SAPS II = simplified acute physiology score II, ICU LOS = intensive care unit length of stay, ICU = intensive care unit, RSBI = rapid shallow breathing index, ΔPocc = end-expiratory airway occlusion pressure, P0.1 = negative airway pressure generated during the first 100 millisecond. Continuous variables are shown as mean (SD), median [IQR] or count (percentage) as appropriate.

eTable 3. The Sensitivity and Specificity of LUSS in Different Cut-Off Values and The Presence of Pendelluft to Predict Liberation Failure.

| MV liberation outcome in validation cohort (n=43) | | |
| --- | --- | --- |
| Cut-off of LUSS to predict liberation failure | Sensitivity | Specificity |
| At the beginning of SBT  LUSS > 9 | 44% | 50% |
| LUSS > 13 | 24% | 83% |
| LUSS > 15 | 16% | 83% |
| LUSS > 18 | 4% | 94% |
| At the end SBT  LUSS > 9 | 54% | 50% |
| LUSS > 13 | 33% | 69% |
| LUSS > 15 | 25% | 81% |
| LUSS > 18 | 8% | 94% |
| MV liberation outcome in both cohorts (n=98) | | |
|  | Sensitivity | Specificity |
| The presence of pendelluft to predict liberation failure | 71% | 55% |

Definition of abbreviations: LUSS = lung ultrasound score, MV = mechanical ventilation, SBT = spontaneous breathing trial

eTable 4. The Number of Patients when LUSS in Different Cut-Off Values and The Presence of Pendelluft Could Predict MV Liberation Outcome but The Absolute Ventral-to-Dorsal Difference Was Misclassified.

|  | MV liberation outcome in validation cohort (n=43) | | | |
| --- | --- | --- | --- | --- |
|  | Success (n=18)  When the absolute ventral-to-dorsal difference > 20 | | Failure (n=25)  when the absolute ventral-to-dorsal difference ≤ 20 | |
|  | At the beginning of SBT | | At the beginning of SBT | |
| The number of patients, n (%) | LUSS ≤ 9 | 3 of 9 (33%) | LUSS > 9 | 3 of 11 (27%) |
|  | LUSS ≤ 13 | 3 of 15 (20%) | LUSS > 13 | 2 of 6 (33%) |
|  | LUSS ≤ 15 | 4 of 15 (27%) | LUSS > 15 | 1 of 4 (25%) |
|  | LUSS ≤ 18 | 4 of 17 (24%) | LUSS > 18 | 1 of 1 (100%) |
|  | At the end of SBT | | At the end of SBT | |
|  | LUSS ≤ 9 | 2 of 8 (25%) | LUSS > 9 | 4 of 13 (31%) |
|  | LUSS ≤ 13 | 3 of 15 (20%) | LUSS > 13 | 2 of 8 (25%) |
|  | LUSS ≤ 15 | 3 of 13 (23%) | LUSS > 15 | 1 of 6 (17%) |
|  | LUSS ≤ 18 | 3 of 15 (20%) | LUSS > 18 | 0 of 2 (0) |
|  | MV liberation outcome in both cohorts (n=98) | | | |
|  | Success (n=40)  when the absolute ventral-to-dorsal difference > 20 | | Failure (n=58)  when the absolute ventral-to-dorsal difference ≤ 20 | |
| The number of patients, n (%) | The absence of pendelluft  4 of 22 (18%) | | The presence of pendelluft  11/41 (27%) | |

Definition of abbreviations: LUSS = lung ultrasound score, MV = mechanical ventilation, SBT = spontaneous breathing trial

eTable 5. Areas Under the Curve (AUC) to Assess the Predictive Capacity in Liberation Outcome.

|  | The AUC of validation cohort for the absolute ventral-to-dorsal difference | The AUC of validation cohort for the absolute ventral-to-dorsal difference plus LUSS | P-value |
| --- | --- | --- | --- |
| At the beginning of SBT | | | |
| LUSS > 9 | 0.76 | 0.51 | 0.003 |
| LUSS > 13 | 0.76 | 0.63 | 0.049 |
| LUSS > 15 | 0.76 | 0.61 | 0.019 |
| LUSS > 18 | 0.76 | 0.64 | 0.027 |
| At the end of SBT | | | |
| LUSS > 9 | 0.79 | 0.54 | 0.011 |
| LUSS > 13 | 0.79 | 0.59 | 0.027 |
| LUSS > 15 | 0.79 | 0.61 | 0.013 |
| LUSS > 18 | 0.79 | 0.68 | 0.046 |
|  | The AUC of the overall population for the absolute ventral-to-dorsal difference | The AUC of the overall population for the absolute ventral-to-dorsal difference plus the presence of pendelluft |  |
| The presence of pendelluft | 0.80 | 0.66 | 0.005 |

Definition of abbreviations: LUSS = lung ultrasound score, AUC = area under the curve

**Supplement References**

1. Franchineau G, Jonkman AH, Piquilloud L, et al. Electrical Impedance Tomography to Monitor Hypoxemic Respiratory Failure. *Am J Respir Crit Care Med*. Dec 21 2023;doi:10.1164/rccm.202306-1118CI

2. Greenblatt EE, Butler JP, Venegas JG, Winkler T. Pendelluft in the bronchial tree. *J Appl Physiol (1985)*. Nov 1 2014;117(9):979-88. doi:10.1152/japplphysiol.00466.2014

3. Soummer A, Perbet S, Brisson H, et al. Ultrasound assessment of lung aeration loss during a successful weaning trial predicts postextubation distress*. *Critical Care Medicine*. 2012;40(7):2064-2072. doi:10.1097/CCM.0b013e31824e68ae

4. Caltabeloti F, Monsel A, Arbelot C, et al. Early fluid loading in acute respiratory distress syndrome with septic shock deteriorates lung aeration without impairing arterial oxygenation: a lung ultrasound observational study. *Crit Care*. May 6 2014;18(3):R91. doi:10.1186/cc13859

5. Mayo PH, Copetti R, Feller-Kopman D, et al. Thoracic ultrasonography: a narrative review. *Intensive Care Medicine*. 2019/09/01 2019;45(9):1200-1211. doi:10.1007/s00134-019-05725-8

6. Mongodi S, Bouhemad B, Orlando A, et al. Modified Lung Ultrasound Score for Assessing and Monitoring Pulmonary Aeration. *Ultraschall Med*. Oct 2017;38(5):530-537. Modifizierter Lungen-US-Score zur Bewertung und Überwachung der Belüftung der Lunge. doi:10.1055/s-0042-120260

7. Longhini F, Maugeri J, Andreoni C, et al. Electrical impedance tomography during spontaneous breathing trials and after extubation in critically ill patients at high risk for extubation failure: a multicenter observational study. *Annals of Intensive Care*. 2019/08/13 2019;9(1):88. doi:10.1186/s13613-019-0565-0

8. Wan X, Wang W, Liu J, Tong T. Estimating the sample mean and standard deviation from the sample size, median, range and/or interquartile range. *BMC Medical Research Methodology*. 2014/12/19 2014;14(1):135. doi:10.1186/1471-2288-14-135
